# Predictors of Changes in Pelvic Rotation after Surgery with or without Femoral Derotational Osteotomy in Ambulatory Children with Cerebral Palsy

**DOI:** 10.3390/bioengineering10101214

**Published:** 2023-10-18

**Authors:** Reiko Hara, Susan A. Rethlefsen, Tishya A. L. Wren, Robert M. Kay

**Affiliations:** Motion and Sports Analysis Laboratory, Jackie and Gene Autry Orthopedic Center, Children’s Hospital Los Angeles, Los Angeles, CA 90027, USA; srethlefsen@chla.usc.edu (S.A.R.); twren@chla.usc.edu (T.A.L.W.); rkay@chla.usc.edu (R.M.K.)

**Keywords:** pelvic rotation, gait analysis, femoral derotational osteotomy, cerebral palsy

## Abstract

Asymmetry of pelvic rotation affects function. However, predicting the post-operative changes in pelvic rotation is difficult as the root causes are complex and likely multifactorial. This retrospective study explored potential predictors of the changes in pelvic rotation after surgery with or without femoral derotational osteotomy (FDRO) in ambulatory children with cerebral palsy (CP). The change in the mean pelvic rotation angle during the gait cycle, pre- to post-operatively, was examined based on the type of surgery (with or without FDRO) and CP distribution (unilateral or bilateral involvement). In unilaterally involved patients, pelvic rotation changed towards normal with FDRO (*p* = 0.04), whereas patients who did not undergo FDRO showed a significant worsening of pelvic asymmetry (*p* = 0.02). In bilaterally involved patients, the changes in pelvic rotation did not differ based on FDRO (*p* = 0.84). Pelvic rotation corrected more with a greater pre-operative asymmetry (β = −0.21, SE = 0.10, *p* = 0.03). Sex, age at surgery, GMFCS level, and follow-up time did not impact the change in pelvic rotation. For children with hemiplegia, internal hip rotation might cause compensatory deviation in pelvic rotation, which could be improved with surgical correction of the hip. The predicted changes in pelvic rotation should be considered when planning surgery for children with CP.

## 1. Introduction

Cerebral palsy (CP) is a neuromotor disorder affecting movement and posture that is usually caused by damage to the developing brain of a fetus or infant [1,2]. Although the etiology of the condition has not been fully understood, CP has been reported to have a prevalence of 1 to nearly 4 per 1000 children in the United States and other parts of the world [2,3,4,5,6,7,8,9,10]. As such, it is considered one of the most common physical disabilities in childhood [2,3,8,9,10]. This condition is highly heterogeneous, and the severity and involvement of the condition vary across individuals. The underlying brain injury is non-progressive, but the neuromuscular and orthopedic manifestations can change over time. The symptoms of CP include difficulty in controlling movement, spasticity and involuntary motions, and stiff muscles; these impairments often lead to secondary problems such as muscle weakness, muscle and joint contracture, or bony deformities. Due to these neuromuscular and musculoskeletal problems, children with CP often experience gait impairment and abnormal walking patterns. Due to the complexity and variability of the underlying etiologies, treatment of these problems is multifaceted, and clinical outcomes can be difficult to predict.

Treatment of gait problems in CP often requires orthopedic surgery to address issues involving multiple muscles, bones, and joints. The State-of-the-Art approach is single-event multilevel surgery, in which all problems are addressed in a single surgical session [11,12]. As multiple issues are being addressed simultaneously, it is important to be able to assess all lower extremity levels (hips, knees, ankles, and feet) in all three anatomic planes (sagittal, coronal, and transverse) to identify gait deviations in need of correction and their primary causes. Since single-event multilevel surgery can have indirect effects, it is also important to study the surgical outcomes, especially with respect to secondary or compensatory movement patterns.

**Figure 1:**
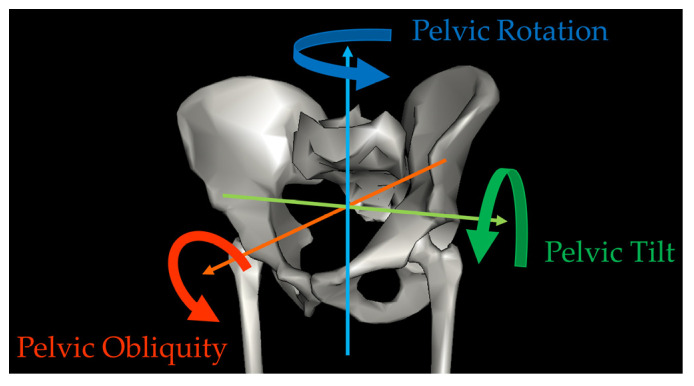
Pelvic kinematics in three-dimensional gait analysis.

Three-dimensional (3D) instrumented gait analysis has routinely been used as a diagnostic tool to assess the state of locomotion and to plan surgical and other treatments for children with CP in the clinical practice of pediatric orthopedics [13,14,15]. Gait analysis provides quantitative information on human body movement, assisting in the identification of problems at each joint level and allowing the planning of single-event multilevel surgery for individual patients [16,17,18]. Using retroreflective markers placed on the skin surface and multiple infrared light cameras to identify the location of the markers over time, the motion capture system and its biomechanical model determine the position of body segments in three dimensions, enabling the calculation of relative motion between segment-fixed coordinate systems (kinematics), including joint angles between adjacent segments, during movement such as ambulation [19]. Kinematics are typically described as rotations around three orthogonal axes representing motion in the sagittal, coronal, and transverse planes. In the case of the pelvis, which is usually the most proximal segment in a lower extremity model, kinematics are expressed relative to a fixed coordinate system representing the laboratory, and motion in the sagittal, coronal, and transverse planes are referred to as pelvic tilt, obliquity, and rotation, respectively (Figure 1).

Rotational deviations in the transverse plane are a common problem that hinder the walking ability of children with CP, resulting in pathologic gait and lever arm dysfunction. Rotational problems also often result in in-toeing in children with CP which can, in turn, cause functional problems such as tripping. Rotational problems can occur at multiple levels in the pelvis, hip, knee, ankle, and foot. Pelvic rotation is one of the significant contributors to rotational deviations. However, the root causes of rotational deviation at the pelvis are complex and likely multifactorial, involving problems at other joints, such as torsional deformities of the leg bones, bone deformities in the feet, muscle imbalance, weakness, tightness or contracture, neuromuscular control impairment, or any combination of these factors [20,21,22,23]. In many cases, abnormal pelvic rotation may occur as a compensation to normalize foot progression (rotational alignment of the foot relative to the forward direction of walking). For example, patients with internal hip rotation and in-toeing may rotate their pelvis outward, where the foot points in a less inward and in a more forward position [22,24]. Pelvic rotation is also associated with clinical measures and gait parameters at the ankle joint, such as tightness of calf muscles, decreased ankle dorsiflexion, and decreased push-off energy, in children with hemiplegic CP [22,25,26].

The complexity of the underlying mechanisms that can cause deviations in pelvic rotation makes it difficult to predict the post-operative changes in the pelvic rotation angle. The change in pelvic rotation is likely to vary based on the surgical procedures performed as well as patient characteristics, such as involvement and functional level. In 2004, Kay et al. retrospectively studied the changes in the pelvic rotation angle after femoral derotational osteotomy (FDRO) and/or soft tissue surgeries in 59 children with CP [20]. Femoral derotational osteotomy is a bony surgical procedure in which the femur is cut transversely, rotated, and fixed in the realigned position to correct the rotational alignment of the femur. This is a common procedure performed in patients with CP who have excessive internal or external hip rotation due to femoral anteversion or retroversion. Soft tissue surgery, on the other hand, refers to procedures such as tendon lengthening and transfer, which do not directly involve bones. The authors found improvement in the pelvic rotation angle after both types of surgery, with changes occurring in both children with unilateral involvement (hemiplegia) and bilateral involvement (diplegia or quadriplegia) [20].

Several other studies have also reported improvement in the pelvic rotation angle after surgery [27,28,29,30,31,32,33,34,35]. Some of these studies compared the effects of surgery with and without FDRO, similar to what Kay et al. examined. However, unlike the results from Kay et al., these studies showed an improvement in pelvic rotation only when FDRO was performed [28,29]. Pelvic rotation did not improve in these studies when only soft tissue surgery was performed. In contrast, other studies found an improvement in the pelvic rotation angle after soft tissue surgery [30,31]. In addition, the change in pelvic rotation may differ based on whether FDRO is performed unilaterally or bilaterally. A study by Niklasch et al., for instance, compared the influence of unilateral and bilateral FDRO in children with bilateral CP who demonstrated over 20 degrees of asymmetry pre-operatively, observing an improvement in the pelvic rotation angle in the unilateral FDRO group but not in the group with bilateral FDRO [33]. 

Although all of the above investigations were conducted on children with CP, characteristics of the examined patients varied between studies. Laterality and involvement could include any of the following: hemiplegia, diplegia, bilateral involvement (diplegia, triplegia, and quadriplegia), or all types of CP. In addition, some studies further classified children by Gross Motor Function Classification System (GMFCS) level, which characterizes the severity of lower extremity impairment [36], or used the pre-operative angle of pelvic rotation. The influence of FDRO on the pelvic rotation angle tends to be most clearly shown in hemiplegic or unilaterally involved patients and patients functioning at a higher level [27,28,29,35]. In fact, a systematic review and meta-analysis by Carty et al. concluded that FDRO improves pelvic rotation in unilateral CP but not in bilaterally involved patients [27]. Similarly, patients who had greater deviation in pelvic rotation pre-operatively showed a greater change in the pelvic rotation angle after surgery [30,34,35]. This is because the mechanisms causing the deviation in pelvic rotation are likely different between hemiplegia and diplegia and across patients, particularly for diplegic children [22,25], and patients might respond differently to surgery based on their involvement and individual characteristics. 

Since the results of previous studies are variable, perhaps due to the diversity of research methods and study population, it is difficult to interpret the disparate results from previous research [27]. Hence, the contributors to the changes in pelvic rotation after surgery remain uncertain. The purpose of this study is to explore potential predictors of the changes in pelvic rotation after surgery with or without FDRO in ambulatory children with CP. This study examines a large cohort including both unilaterally and bilaterally involved patients who had surgery with or without FDRO. Understanding when and to what extent pelvic rotation is likely to change can help surgeons design surgical plans to maximize post-operative function.

## 2. Materials and Methods

Approval from the institutional review board (IRB) at our hospital was granted prior to the commencement of this study. Patients who underwent gait analysis in our motion analysis laboratory before 31 December 2018 were covered under a retrospective IRB approval with a waiver of consent. For patients who were seen after that date, written consent and assent were obtained at the time of clinical gait analysis for their data to be used for research.

### 2.1. Study Participants

Ambulatory patients with CP who underwent lower extremity orthopedic surgery with or without an FDRO procedure at our institution between 1 January 2005 and 26 October 2022, who had pre- and post-operative gait analysis, were included in this retrospective study. Cases with concomitant tibial derotational osteotomy to correct excessive internal or external rotation of the tibia or surgical procedures to correct rotational alignment of the foot which could affect foot progression (i.e., calcaneal sliding osteotomy, posterior tibialis tendon lengthening, split anterior tibialis tendon transfer, etc.) were excluded to isolate the impact of FDRO on the changes in the pelvic rotation angle. For patients who had multiple surgeries with more than two gait analyses, the first qualified surgery and the associated pre- and post-operative gait analysis were used. 

### 2.2. Gait Analysis Data Collection and Processing

All of the data were originally collected and processed using a Vicon Motion Capture System (Vicon Motion Systems Ltd., Yarnton, Oxford, UK). The data collected before 11 February 2015 used an 8 camera Workstation system. The data collected after 11 February 2015 utilized a 10 camera Nexus 2 system. Both systems used the same gait model, and the data are considered equivalent between the two systems. All of the data collection was performed in accordance with the standard procedures for clinical gait analysis at our laboratory. These procedures and the processes for quality assurance were accredited by the Commission for Motion Laboratory Accreditation. To track the patient’s motion, retroreflective markers were placed on the skin surface following a modified version of the Plug-in Gait model (Vicon Motion Systems Ltd., Yarnton, Oxford, UK). Plug-in Gait is the Vicon implementation of the most commonly used gait analysis model, termed the “conventional gait model”. Two modifications were made, which have been shown to improve the data accuracy. First, to improve tracking of the thigh segment, a marker on the center of the patella was used instead of the traditional thigh “wand” which uses a marker at the end of a short stick attached to the lateral thigh [37]. Second, to improve tracking of the shank segment, a marker placed anteriorly on the tibial crest was used instead of a tibial wand marker on the lateral shank [38,39].

During the data collection, patients walked barefoot across a 15 m walkway in the laboratory multiple times at a self-selected speed with all necessary markers attached on their skin surface. The motion capture system recorded and triangulated the marker locations to define the position and orientation of rigid segments in a linked-body model. The segments defined were the pelvis and left and right thighs, shanks, and feet. Pelvic orientation was defined relative to the laboratory using Euler rotations in a rotation–obliquity–tilt rotation sequence [40]. Positive values for pelvic rotation indicate internal (or forward) rotation in the transverse plane while negative values indicate external (or outward) rotation. All kinematics were calculated and recorded at every 2% of the gait cycle, starting from initial contact when a foot first contacted the ground (0%) to the next initial contact of the same foot (100%), providing 51 data points per gait cycle for each side.

The gait data were extracted retrospectively using Visual 3D (C-Motion, Inc., Boyds, MD, USA). An average of several representative strides for each patient and visit was computed. Mean pelvic rotation angle during the gait cycle was then calculated as an average of the 51 data points for the average gait cycle. This variable indicates overall rotational positioning of the pelvis and was the main outcome measure utilized for the analysis. In typical gait for persons without disability, the mean pelvic rotation is close to zero, indicating symmetry of motion. For each patient, the data from the trailing side (more externally rotated side pre-operatively) was used in the analysis. The change in the pelvic rotation angle was defined as the difference in the angle between the pre- and post-operative sessions, in which positive values indicate the correction of the pelvic rotation angle towards a neutral/normal position.

### 2.3. Statistical Analysis

The change in the pelvic rotation angle was assessed based on the type of surgery (with or without FDRO) and the distribution of CP (unilateral or bilateral involvement). The FDRO group was comprised of patients who underwent a lower extremity orthopedic surgery that included FDRO (with or without other procedures to address soft tissues), while patients in the No FDRO group had surgery that did not include the FDRO procedure. Patients were also classified, based on the distribution of CP, into two groups with unilateral involvement (left or right hemiplegia) or bilateral involvement (diplegia, triplegia, or quadriplegia). The analysis was stratified with the CP distribution to analyze the unilaterally involved and bilaterally involved groups separately.

Linear regression analysis examined whether having an FDRO affected the change in the pelvic rotation angle within each group. Other potential predictors of the changes in pelvic rotation were also examined using univariate linear regression including sex, age at surgery, GMFCS level, time since surgery, and pre-operative pelvic rotation angle. The significance level was set at 0.05. Predictors significant at the 0.05 level were then included in multivariate analysis as appropriate. Statistical analyses were performed with Stata 14.2 (StataCorp LLC., College Station, TX, USA).

## 3. Results

This study cohort included 101 patients whose mean age was 10.2, with a standard deviation (SD) of 3.6 years. A total of 63 of 101 (62%) patients were male. Distribution of the GMFCS level at the pre-operative visit was 20, 46, 30, and 5 (20%, 46%, 30%, and 5%) for GMFCS I–IV, respectively. A total of 17 (17%) patients had unilateral CP involvement, and 84 (83%) had bilateral involvement. A total of 18 (18%) patients underwent unilateral FDRO, 39 (39%) received bilateral FDRO, and 44 (44%) had surgery without FDRO. All FDROs were external to correct internal hip rotation. The characteristics of patients and their surgical treatments are summarized in Table 1.

**Figure 2:**
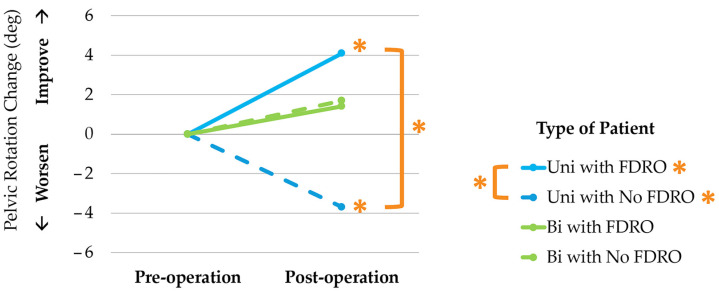
Changes in the pelvic rotation angle with or without FDRO in patients with unilateral (Uni) and bilateral (Bi) involvement. * Shows a statistical significance within and between groups at *p* < 0.05.

In unilaterally involved patients, the post-operative change in the pelvic rotation angle demonstrated a significant difference between patients who underwent FDRO and those who had surgery without FDRO (*p* = 0.004, Table 2), in addition to there being significant changes within each of the groups pre- to post-operatively. For those who underwent FDRO, the predicted mean ± standard error of the change in the pelvic rotation angle after surgery was an improvement towards a normal angle of 4.1 ± 1.8 degrees (*p* = 0.04), while those who did not undergo FDRO had a significant worsening of pelvic asymmetry with a predicted mean change of −3.7 ± 1.5 degrees (*p* = 0.02, Table 2). The results are depicted in Figure 2. The change in the pelvic rotation angle was not affected by the other predictors, which included sex, GMFCS level, age at surgery, follow-up time, and the pre-operative pelvic rotation angle for unilaterally involved patients (*p* > 0.33, Table 3).

For bilaterally involved patients, the post-operative pelvic rotation angle improved slightly regardless of whether FDRO was performed; however, the changes were not statistically significant (Table 2, Figure 2). The predicted mean change was 1.4 ± 0.8 degrees (*p* = 0.08) for those who underwent FDRO and 1.7 ± 1.0 degrees (*p* = 0.09) for those who did not receive FDRO. Therefore, the post-operative change in pelvic rotation did not differ significantly based on the procedure of FDRO in bilaterally involved patients (*p* = 0.84). Of note, these results were not affected by whether the FDROs were unilateral or bilateral (*p* = 0.94). The pre-operative pelvic rotation angle was found to be a significant predictor of the post-operative changes in pelvic rotation for bilaterally involved patients (*p* = 0.03, Table 3); however, sex, GMFCS level, age at surgery, and time since surgery did not have a significant impact on the changes in the pelvic rotation angle (*p* > 0.07). Since only one significant predictor of the change in pelvic rotation was found for each subgroup (FDRO for unilaterally involved patients; pre-operative pelvic rotation for bilaterally involved patients), multivariable analysis was not performed.

## 4. Discussion

This retrospective study investigated factors that may affect the change in pelvic rotation after surgery in children with CP. For unilaterally involved patients, a significant effect of the procedure of FDRO on the post-operative change in the pelvic rotation angle was confirmed both within and between groups; patients demonstrated an improvement in the pelvic rotation angle with FDRO and worsening of the angle without FDRO. For bilaterally involved patients, the pelvic rotation angle improved slightly with or without FDRO, and no influence of the procedure on the pelvic rotation angle was apparent. Although other potential predictors such as sex, GMFCS level, age at surgery, follow-up time, and the pre-operative pelvic rotation angle failed to show an impact on the post-operative change in the pelvic rotation angle in unilaterally involved children, an association between the pre-operative pelvic rotation angle and the post-operative change of the angle was evident in bilaterally involved patients. This suggests that lower extremity surgery, in general, tends to improve pelvic rotation in bilaterally involved patients who start with greater pelvic rotation asymmetry.

Our results indicated that the FDRO procedure had a significant impact on the post-operative change in the pelvic rotation angle for patients with hemiplegic CP. Their pelvic rotation improved when FDRO was performed, while pelvic asymmetry became worse when FDRO was not performed. An improvement in pelvic rotation after FDRO in unilaterally involved patients has previously been reported [27,28]. Our results also revealed worsening of pelvic rotation asymmetry in hemiplegic patients who had surgery without FDRO, which, to our knowledge, has not been previously recognized. It is possible that some unilaterally involved patients did not receive FDRO even though the treatment was indicated, and their pelvic rotation, as a result, may have worsened over time.

All FDRO procedures in this study were external in direction to correct excessive internal hip rotation. For patients with hemiplegia, internal hip rotation might be a cause of compensatory outward deviation in pelvic rotation to normalize the foot progression angle. This compensatory pelvic rotation is improved with surgical correction of the hip rotation. On the other hand, for hemiplegic patients who did not need hip rotation correction with FDRO, pelvic rotation asymmetry may have been associated with other factors, which resulted in worsening of the condition after surgery. 

For patients with bilateral involvement, pelvic rotation showed a small correction towards neutral, whether or not FDRO was performed, suggesting that their deviation in pelvic rotation might be rooted in factors other than internal hip rotation. An improvement in pelvic rotation after soft tissue surgery has previously been reported by several studies [20,30,31]. Children with bilateral involvement have a wider variety of conditions, and the heterogeneity among individuals may make the causes of deviation in pelvic rotation more complicated. Consequently, the outcome of surgery is harder to predict in bilaterally involved patients.

The pre-operative pelvic rotation angle showed a significant influence on the post-operative change in the pelvic rotation angle for bilaterally involved patients, which is consistent with the previous literature [30,34,35]. Therefore, a greater pelvic asymmetry before surgery appears to be a good indicator of a potential improvement in pelvic rotation after surgery. On the other hand, since no association was observed between other potential predictors (age, sex, GMFCS level, and time since surgery) and the change in pelvic rotation, post-operative pelvic rotation might not be affected by these more general characteristics. Instead, the rotational profile of each individual, including the need for FDRO (in unilaterally involved patients) and the pre-operative pelvic rotation angle (in bilaterally involved patients) would provide a better prediction of the post-operative pelvic rotation angle.

The changes in pelvic rotation are generally a secondary effect of surgery rather than a direct target of the surgical intervention. FDRO is performed to correct rotational alignment at the hip but also affects pelvic rotation and the foot progression angle [35,41]. Foot surgery also influences compensatory gait deviations in patients with CP, including an improvement in pelvic rotation after surgery [42]. Due to the complexity and diversity of the underlying problems, the gait deviations in patients with CP do not occur at a single joint level. Instead, they involve multi-segmental levels which influence each other. The knowledge of the predicted outcomes of multilevel surgery with respect to the entire lower extremities plays an essential role in surgical decision making. Investigations to elucidate the influences of different treatment and patient-related factors are, therefore, crucial to maximizing outcomes.

More specifically, the clinical motivation for studying the changes in pelvic rotation is to optimize surgical outcomes by optimizing treatment planning. If the expected post-operative change in pelvic rotation can be predicted, this change can be accounted for during surgical planning. For example, the amount of derotation to be performed during FDRO might be adjusted for the expected change in pelvic rotation. If an improvement in pelvic rotation occurs but was not accounted for during surgical planning, the limb will be under corrected. Given the difference in the expected change of pelvic rotation between unilaterally and bilaterally involved patients, our results suggest that a unilaterally involved patient should undergo greater derotation than a bilaterally involved patient with similar pre-operative deformity. In addition, if one of the main motivators for FDRO in a patient with bilateral CP is pelvic asymmetry, it does not appear that bilateral FDRO accomplishes this any better than unilateral FDRO.

The current investigation focused on the impact of FDRO, excluding other bone procedures commonly performed to correct the rotational alignment at lower extremity levels. While this allowed us to better isolate the effects of FDRO on the changes in pelvic rotation, it is anticipated that the post-operative pelvic rotation can also be affected by other surgical procedures at different joint levels. The impact of such procedures, including tibial rotational osteotomy, needs to be assessed in future studies for a more complete understanding of the changes in the post-operative pelvic rotation following single-event multilevel surgery.

### Limitations

The data in this study were limited to those from a single motion laboratory at a single institution, situated in a large metropolitan city. Even though our patient population includes a variety of races and ethnicities, the generalizability of the results might still be considered as a limitation. Another limitation of this investigation was the retrospective nature of the study design: it was not possible to control the variables in terms of age at surgery, length of time from pre-operative gait analysis to surgery, as well as from surgery to post-operative gait analysis. Although those variables and their impacts were considered in statistical analysis, it is possible that other factors besides surgery might have influenced patients’ post-operative gait parameters. For instance, some patients had a very long term of a follow up (over 10 years) between surgery and the post-operative gait analysis. The changes associated with growth (i.e., increase in body size, the change in muscle strength, or the change in joint flexibility) might have had an impact on the results. It would be important to investigate the isolated impact of surgery with controlled periods of follow ups as well as the long-term influence of surgery and/or growth on the changes in pelvic rotation in the future.

## 5. Conclusions

This study examined potential predictors of the changes in pelvic rotation after surgery with or without FDRO in ambulatory children with CP. Although FDRO is primarily performed to correct rotational malalignment at the hip, the results suggested that the procedure also influenced pelvic rotation post-operatively. For children with unilateral CP, surgery including FDRO resulted in an improvement in pelvic asymmetry, whereas surgery without FDRO resulted in worsening of pelvic rotation. For children with bilateral CP, post-operative change in pelvic rotation was smaller and was not affected by FDRO, although a larger improvement could be expected in patients who had greater pelvic deviation pre-operatively. Rotational profiles at the pre-operative visit and the predicted changes in pelvic rotation should be considered when planning surgical interventions for children with CP.

## Figures and Tables

**Table 1:** Characteristics of patients (N = 101).

Predictive Variables	Number (%) of Patients in Each Group or Mean ± Standard Deviation (Range)
Distribution of CP	
Unilateral Involvement	17 (17%)
Bilateral Involvement	84 (83%)
Type or Surgery	
No FDRO	44 (44%)
Unilateral FDRO	18 (18%)
Bilateral FDRO	39 (39%)
Sex	
Male	63 (62%)
Female	38 (38%)
GMFCS Level	
I	20 (20%)
II	46 (46%)
III	30 (30%)
IV	5 (5%)
Age at Surgery (years)	10.2 ± 3.6 (4.8 – 20.7)
Follow-up Time (years)	2.4 ± 2.4 (0.6 – 12.3)
Pre-operative Pelvic Rotation Angle (degrees)	−6.1 ± 6.2 (−35.2 – 1.1)

Categorical variable: number (%) of patients in each group. Continuous variable: mean ± standard deviation (range).

**Table 2:** Change in pelvic rotation angle based on CP distribution and status of FDRO.

Classification of Patients	Change in Pelvic Rotation Angle (Deg)	*p*-Value Change in Pelvic Rotation Angle	*p*-Value FDRO vs. No FDRO
Unilateral Involvement			0.004
FDRO (*n* = 7)	4.1 (1.8)	0.04	
No FDRO (*n* = 10)	−3.7 (1.5)	0.02	
Bilateral Involvement			0.84
FDRO (*n* = 50)	1.4 (0.8)	0.08	
No FDRO (*n* = 34)	1.7 (1.0)	0.09	

Positive values of the change in angle indicate correction of pelvic rotation towards normal. Results are presented as model-predicted mean (standard error).

**Table 3:** Impact of other predictors on the changes in the pelvic rotation angle.

Predictors	*p*-Value Unilateral Involvement	*p*-Value Bilateral Involvement
Sex	0.51	1.00
GMFCS Level	0.73	0.07
Age at Surgery	0.33	0.66
Follow-up Time	1.00	0.82
Pre-operative Pelvic Rotation Angle	0.69	0.03 *

* Coefficient = −0.21 and standard error = 0.10.

## Data Availability

This study’s data are not publicly available due to restrictions for the data sharing by the Institutional Review Board at the authors’ institution. However, the data will be made available from the corresponding author upon reasonable request.
